# Application of Graph-Theoretic Methods Using ERP Components and Wavelet Coherence on Emotional and Cognitive EEG Data

**DOI:** 10.3390/brainsci15070714

**Published:** 2025-07-02

**Authors:** Sencer Melih Deniz, Ahmet Ademoglu, Adil Deniz Duru, Tamer Demiralp

**Affiliations:** 1 Institute of Biomedical Engineering, Bogazici University, Istanbul 34684, Turkey; 2 The Scientific and Technological Research Council of Turkey (TÜBITAK) Informatics and Information Security Research Center (BILGEM), Kocaeli 41400, Turkey; 3 Faculty of Sports Science, Marmara University, Istanbul 34722, Turkey; 4 Department of Physiology, Istanbul Faculty of Medicine, Istanbul University, Istanbul 34093, Turkey; 5 Hulusi Behcet Life Sciences Research Laboratory, Neuroimaging Unit, Istanbul University, Istanbul 34093, Turkey

**Keywords:** EEG, event-related potentials, wavelet coherence, brain–computer interface, emotion, cognition, classification

## Abstract

**Background/Objectives:** Emotion and cognition, two essential components of human mental processes, have traditionally been studied independently. The exploration of emotion and cognition is fundamental for gaining an understanding of human mental functioning. Despite the availability of various methods to measure and evaluate emotional states and cognitive processes, physiological measurements are considered to be one of the most reliable methods due to their objective approach. In particular, electroencephalography (EEG) provides unique insight into emotional and cognitive activity through the analysis of event-related potentials (ERPs). In this study, we discriminated pleasant/unpleasant emotional moods and low/high cognitive states using graph-theoretic features extracted from spatio-temporal components. **Methods:** Emotional data were collected at the Physiology Department of Istanbul Medical Faculty at Istanbul University, whereas cognitive data were obtained from the DepositOnce repository of Technische Universität Berlin. Wavelet coherence values for the N100, N200, and P300 single-trial ERP components in the delta, theta, alpha, and beta frequency bands were investigated individually. Then, graph-theoretic analyses were performed using wavelet coherence-based connectivity maps. Global and local graph metrics such as energy efficiency, strength, transitivity, characteristic path length, and clustering coefficient were used as features for classification using support vector machines (SVMs), k-nearest neighbor(K-NN), and linear discriminant analysis (LDA). **Results:** The results show that both pleasant/unpleasant emotional moods and low/high cognitive states can be discriminated, with average accuracies of up to 92% and 89%, respectively. **Conclusions:** Graph-theoretic metrics based on wavelet coherence of ERP components in the delta band with the SVM algorithm allow for the discrimination of emotional and cognitive states with high accuracy.

## 1. Introduction

The field of cognitive neuroscience is enriched with electrophysiological studies that use strict experimental designs to examine fundamental cognitive processes such as decision-making, memory, and attention. However, research specifically targeting the neurophysiological assessment of emotions is relatively new, and the results are less conclusive. EEG measurement is widely used as a non-invasive technique that represents an essential electrophysiological tool for researchers investigating the complex relationship between brain activity and various cognitive/emotional processes. It plays a major role in the assessment of motor function, cognitive workload, attention levels, brain and sleep disorders, and emotional evaluations [1]. The increasing demand for effective and reliable techniques to facilitate human–computer interaction underlines the importance of automatic emotion recognition, which has a significant influence on an individual’s cognitive state and is vital for human communication [2,3]. Additionally, emotion recognition is essential for enhancing caregiving via EEG-based brain–computer interfaces (BCIs).

Therefore, EEG-based BCI systems for emotion recognition are receiving increasing attention, particularly among neuroscientists, due to their critical importance in various interdisciplinary applications [4], including cognitive workload estimation [5,6,7], fatigue detection during driving [8], emotion recognition [9,10,11,12,13], and determination of working memory capacity [14]. Various methods are employed to track emotion recognition, including the assessment of startle responses, behavioral responses, autonomic measurements, audio–visual expressions, gestures, neurophysiological parameters, and self-reports [15]. Personal habits shaped by various cultural and language backgrounds have an influence on audio–visual expressions and gestures. On the other hand, physiological signaling techniques such as electroencephalography (EEG), electrocardiography (ECG), and electromyography (EMG) provide more objective and robust outcomes [4,16]. Among the abovementioned methods, only neurophysiological measurements can directly reflect the fundamental structures of the brain, enabling the detection of a wide spectrum of emotional-state dynamics [17]. The exploration of the relationships between EEG signals and emotions receives significant research attention due to the suggestion that frontal-brain electrical activity is linked to the experience of both positive and negative emotions [18]. Recently, affective computing has become a major research direction in the assessment of psychological and psychiatric conditions in individuals with emotional and neurological disorders, as well as in healthy subjects. It represents an emerging technology that integrates emotion into BCI systems [19]. Emotion recognition, whereby subjects’ emotional states are estimated based on their behavioral and physiological responses, is a key goal in affective computing. Hence, research in this field seeks to improve computer intelligence by developing affective user interfaces for healthy individuals, in addition to enhancing the quality of psychiatric healthcare. Emotional states significantly influence decision-making and problem-solving skills. The recognition of emotions can boost emotional intelligence, thereby enhancing both professional and personal performance [20]. Emotion, personality, and motivation are connected with decision-making, which is a complex cognitive process [21,22,23].

Cognitive workload is a primary concept for the evaluation and monitoring of human performance during cognitive tasks [24] and is defined as the mental effort or attention needed to complete a task [25]. Cognitive state is defined in terms of how much cognitive workload a task consumes. A low cognitive state denotes that cognitive demands are minimal, whereas high cognitive states correspond to conditions where an individual engages a substantial amount of cognitive resources to meet task demands effectively. Furthermore, mental health and cognitive workload can be optimized through emotional self-awareness [26]. The measurement of users’ cognitive workload is attracting increasing attention as non-invasive, wearable electrophysiological systems are becoming more prevalent [27]. Memory, attention, language, problem-solving, and planning are examples of cognitive processes that can be monitored using physiological signals in order to enhance evaluations in human–machine interaction systems. Some examples reported in the literature of how EEG-based cognitive workload monitoring can be applied include air traffic management tasks [28,29], the optimization of working conditions and the measurement of cognitive states during office work [30,31], and n-back tasks to measure memory [32,33,34]. In summary, the classification of different cognitive tasks receives extensive coverage in the literature [35,36,37,38]. In this study, both emotional states and cognitive workload were investigated through the analysis of single-trial EEG signals, which are also called event-related potentials (ERPs). EEG signals can be examined in the time domain, in the frequency domain, or in a combination of both. A frequently used method to identify temporal features in the time domain is the measurement of ERPs [17,39]. ERP analysis is conducted in the time domain by inspecting the amplitudes and latencies of major peaks in the averaged waveforms. In frequency-based analysis, Fourier transform, although it provides relevant frequency-based information from stationary EEG segments, does not reveal the timing of transient neural events [40,41]. To address this issue, signal analysis is generally preferred in both the time and frequency domains [41]. Because of the constraints associated with performing an analysis in only a single domain [40,41], signal analysis using wavelet transform in both the time and frequency domains is usually advantageous for decomposing EEG signals into oscillatory components across various frequency bands [42].

Recent advances in computing have made single-trial EEG analysis feasible for real-time feature extraction [17]. Currently, single-trial EEG data classification is widely used in neuroscience to identify cognitive states and human intentions [43]. EEG-based BCIs measure emotions by utilizing the ERP components of brain signals occurring at latencies of approximately 100, 170, and 230–270 ms following stimulus presentation [44,45,46,47]. Studies indicate that the N170 and N200 components can be used to differentiate levels of intensity in facial emotions [47]. Some studies utilize multiple ERP components instead of focusing on a single one [48], since different combinations of ERP components may provide complementary information for discrimination [49]. Recently, it has been confirmed that ERP components N100, P100, N170, N200, N300, and P300 are sensitive to the processing of emotional stimuli [44,45,46,50]. In another study on affective processing, ERPs were calculated by averaging electrodes in certain parts of the brain, specifically the left anterior (FP1, F3, F7, FC5, and T7), right anterior (FP2, F4, F8, FC6, and T8), left posterior (C3, CP5, P3, P7, and O1), and right posterior (C4, CP6, P4, P8, and O2) parts [51]. In addition to emotional conditions, attention and task-related factors are also studied using time-frequency analysis and ERP measurements [52,53,54,55]. The measurement of ERPs offers the ability to continuously assess human processing, enabling the identification of the various stages (such as attentional, cognitive, or perceptional stages) involved in performing an event [56]. Therefore, ERP components were utilized as features for the classification of emotion and cognitive data in this study.

Wavelet analysis can be used to inspect EEG results by decomposing them into oscillatory components across different frequencies as a function of time [42,57], offering an effective method for analyzing transient ERP signals, which are non-stationary in nature [58,59]. Wavelet analysis has been applied in a number of emotional EEG studies [60]. The gamma band has been suggested as a suitable target for EEG-based emotion classification when emotional still images are used as stimuli [60]. The delta, theta, and alpha bands can provide complementary information for the reading of resting-state emotions. The beta and gamma bands are appropriate for reading task-evoked emotions due to their association with mental activities [4]. Therefore, a variety of frequency bands were investigated in this study. Time-frequency representations such as spectrograms, Hilbert–Huang spectra, and the Zhao–Atlas–Marks transform have been used to distinguish between ratings of liking and consciousness [61]. Differential entropy has been explored as a feature for calculating the success rate in the discrimination of positive, negative, and neutral emotional states [62]. Although many features, including differential entropy [63], statistical features [64], and wavelet features [65], are obtained from individual channels, only a limited number are computed across multiple channels to capture inter-channel dependencies [66], such as the asymmetry of power spectral density between two hemispheres [62] and functional connectivity [67].

A key feature of brain signals is the presence of connections among signals from different regions, which is referred to as connectivity [68]. Functional connectivity provides a means of determining whether certain regions in the brain interact with each other [69]. In connectivity-based studies, electrophysiological data such as functional Magnetic Resonance Imaging (fMRI), EEG, and magnetoencephalography (MEG) data are widely used [70]. In other studies, functional connectivity is employed to derive features from multiple channels [67,71]. Various methods are used to understand the functional relationships among different anatomical regions of the brain [72]. Graph theory is applied to analyze functional connectivity based on electrophysiological data [73] in connectivity studies in order to examine the organization of network patterns [74]. Graph theory offers a robust and effective method of multi-dimensional electrophysiological data analysis, as it considers both the spatial and functional dependencies among brain regions [75]. Graph-theoretic metrics allow for the characterization of the stationary behavior of EEG signals in cases where simple linear methods alone have insufficient explanatory power. In this study, various global and local graph metrics were applied to discriminate pleasant/unpleasant emotional moods, in addition to low/high cognitive states.

Traditional EEG signal processing methods focus on analyzing characteristics in both the time and frequency domains in order to explore neural dynamics while failing to consider the detailed spatial relationships among different brain regions. Brain regions that are in close proximity often show similar signaling patterns due to the volume conduction effect. Furthermore, the activities belonging to various sites are generally interconnected, and their relationships can be characterized using metrics such as correlation, synchronization, and coherence [76]. The connectivity information captured by these metrics, in addition to mutual information [16] and phase-locking values (PLVs) [77,78], can be depicted using graphs [72]. However, wavelet coherence offers significant benefits in connectivity analysis compared to conventional correlation or mutual entropy techniques, as it captures the dynamic and non-stationary characteristics of signals by providing time-localized frequency information [79]. Furthermore, window lengths are adjusted in the wavelet coherence method, yielding shorter time windows for high frequencies and longer time windows for low frequencies. This is essential for EEG signals, given that interactions commonly take place in short bursts [80]. Graph signal processing is suitable for analyzing signals with irregular structures, enabling signals to be described and processed using graph vertices [81]. Modeling using graph signals is well suited for evaluating EEG signals with a low spatial resolution, high dimensionality, and irregular structure [82]. Representing EEG signals in this way permits the exploration of spatial relationships among different sites of the brain. Although numerous studies have examined BCI-based emotion recognition, primarily utilizing single-channel-based feature extraction methods, the functional connectivity networks in the brain that are associated with emotion remain largely unexamined [83]. While some research has studied only emotional data, others studies have focused on cognitive data for analysis [84]. Several studies have explored the effectiveness of brain network indices in emotion recognition [85]. In the literature, time-domain [86,87], frequency-domain [88], and time frequency-domain [89,90] analyses have been used to extract features. One study [91] investigated the cognitive process of emotion based on graph theory, while a vast majority of brain network studies have utilized the phase-locking value (PLV) [77,78] or mutual information [16]. On the other hand, ERP components have been mostly utilized as time domain features [92,93,94]. The abovementioned did not use wavelet coherence in a graph-theoretic context and employed the ERP components all together. Furthermore, established benchmarks for a single pipeline that address both emotional and cognitive paradigms are also missing. In this context, our study addresses these gaps by adopting a graph-theoretic approach that utilizes wavelet coherence-based connectivity patterns of various ERP components in both emotional and cognitive settings.

Research indicates that emotional states significantly affect cognitive processes like attention, memory, and decision-making [2,3]. The integration of cognitive and emotional data in EEG-based research is gaining recognition for its importance across multiple fields, especially in BCIs. EEG technology enables researchers to monitor real-time brain activity associated with emotions or cognitive functions. By investigating both emotional and cognitive data, researchers can understand how emotional states affect cognitive performance during BCI tasks. Furthermore, the combination of these data types can result in the creation of BCIs that are more responsive to users’ mental states, enhancing both usability and effectiveness. Therefore, the integration of emotional and cognitive data into EEG research is essential in deepening our understanding of brain function, which enhances BCI technologies. We emphasize that a BCI system for practical applications has to monitor both dimensions at the same time. Therefore, we aimed to benchmark our proposed method on two tasks, namely emotional and cognitive tasks, instead of asserting a causal link. We regard this integration as a promising area for future research, especially on rapidly growing wearable technology, as examining these two dimensions together presents opportunities for innovative applications in BCI technology.

As a novel approach, we propose a pipeline that computes graph-theoretic metrics of connectivity matrices obtained from the wavelet coherence of single-trial N100, N200, and P300 ERP components. These graph-theoretic metrics were used as input features for commonly used classification algorithms. The technique was applied to both emotional and cognitive EEG data to identify pleasant/unpleasant emotional moods, as well as low/high cognitive states.

## 2. Materials and Methods

### 2.1. Emotional Data: Subjects, Data Acquisition, and Experimental Paradigm

This study included thirteen healthy subjects with an average age of 27.4 years (±2.96). The subjects were informed about the study, and their signed consent was obtained before the experiments were conducted. EEG data were collected from 32 channels using a BrainAmp (Brain Products GmbH, Munich, Germany) amplifier at the Physiology Department of the Istanbul Medical Faculty at Istanbul University. The data collection procedure received approval from the Local Ethical Committee of the Istanbul University Faculty of Medicine.

**Figure 1:**
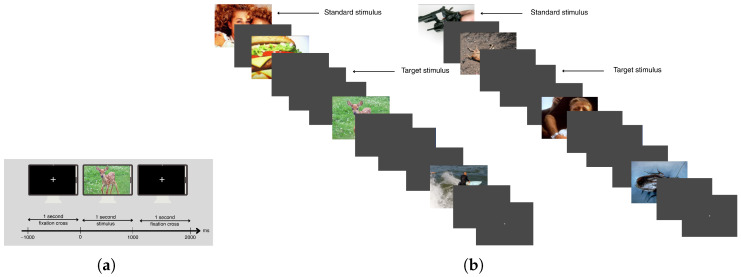
(**a**) Stimulus durations. (**b**) Emotional stimuli.

The subjects were seated in front of a screen (1920 × 1080 resolution LCD monitor) showing visual stimuli in a softly illuminated room designed to isolate sound and electromagnetic interference. They were instructed to focus their attention on the center of the screen during the experiment. The stimuli were displayed one meter away from the subjects, with each stimulus appearing on the screen for one second. The interval between stimuli was set to 2 s, as illustrated in Figure 1.

The experiment employed a modified oddball paradigm to keep the subjects’ attention focused on the stimuli under the passive condition. In two separate sessions, 280 pleasant and 280 unpleasant pictures with positive or negative emotional valence served as standard stimuli. Additionally, a neutral stimulus was randomly introduced as a target stimulus.

**Figure 2:**
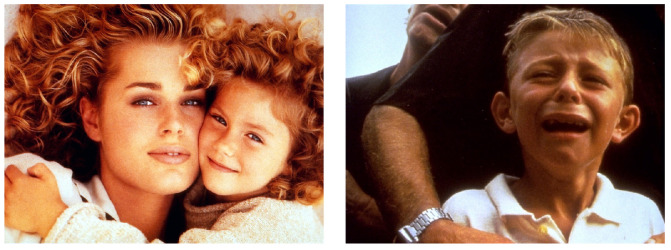
Pleasant (**left**) and unpleasant (**right**) images [95].

Emotional stimuli, chosen for their irrelevance to the task, were used to isolate the effect of emotion on ERPs. Pictures from the IAPS dataset [95] were selected in such a way that the mean valence levels (7.13/2.96) differed but the mean arousal levels (4.99/5.02) remained constant across the two sessions. For half of the subjects, the session began with pleasant pictures, while for the other half, the session began with unpleasant pictures in order to reduce order-related effects. These two sessions are represented in Figure 1. Representative stimuli illustrating the pleasant and unpleasant image categories are presented in Figure 2.

The EEG data were recorded in a unipolar manner, with reference to the mean values of both ear lobes from 30 Ag/AgCl electrodes placed at the FP1, FP2, F3, Fz, F4, FC3, FCz, FC4, F7, F8, FT7, FT8, C3, Cz, C4, CP3, CPz, CP4, T7, T8, P3, Pz, P4, TP7, TP8, P7, P8, O1, Oz, and O2 channels, arranged according to the international 10–20 system. EOG measurements were taken using electrodes positioned on the nasal canthus and the outer canthus of the right eye in order to track eye movements.

### 2.2. Cognitive Data: Subjects, Data Acquisition, and Experimental Paradigm

Cognitive data were obtained from 15 healthy subjects aged 22 to 35, none of whom had acute or chronic neurological or psychiatric disorders or were pregnant women. Eleven of these subjects were right-handed, and ten were male. The data were collected at Technische Universität Berlin, and the study received approval from the university’s Ethical Review Board [96,97]. A BrainAmp amplifier with 64 active electrodes (Brain Products GmbH, Munich, Germany) set up according to the 10–20 international system was used to collect EEG data. An EOG electrode was also placed beneath the left eye to monitor eye movements. Unipolar recording was conducted with a sampling rate of 1 kHz, with the ground electrode positioned at AFz on the scalp and the reference electrode positioned on the left mastoid. The data acquisition system was subsequently re-referenced to both the left and right mastoids.

**Figure 3:**
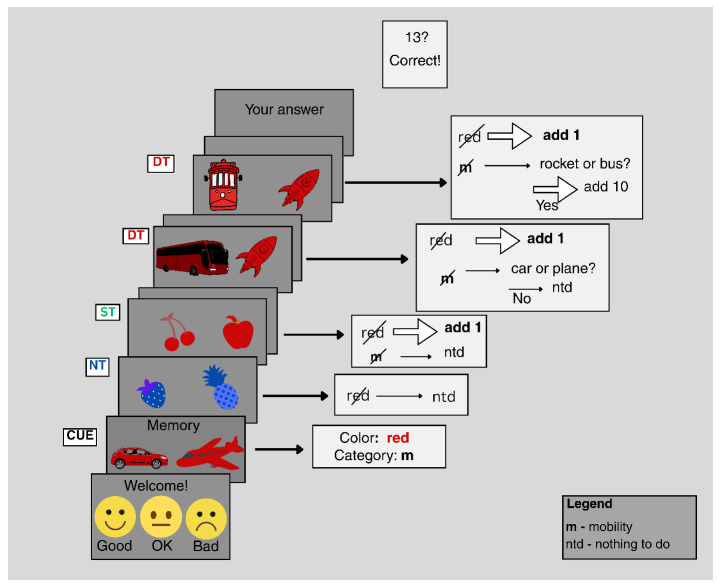
From left to right, assessments of memory (recall memory and compare with the last target pair), language (compare the number of syllables), and visual imagination (imagine the objects in reality and compare the dimensions) [96].

The fifteen subjects are seated in front of a monitor (a 24-inch Dell U2410 display with a 60 Hz refresh rate and a 1920 × 1200 resolution) and were shown a series of visual stimuli, including cartoon depictions of fruits, animals, and transportation vehicles, while engaging in particular tasks linked to each type of stimulus, as shown in Figure 3.

Three levels of cognitive processing were examined: no processing, shallow processing (involving distinguishing stimuli based on color), and deep processing (entailing engagement in a complex activity related to cognitive processing). Each visual stimulus was composed of two cartoon images depicting an object from one of three categories: animals, fruits, or vehicles. These images were presented in one of four colors: red, green, blue, or magenta. The two images shared the same color and belonged to the same category, with each category containing a total of 10 objects [96]. For each run, only two of the three categories were selected in order to maintain the desired ratio and to avoid making the tasks too difficult. Before the actual tests were carried out, each subject underwent 1–3 practice runs to allow them to become accustomed to the tasks.

In the experimental scenario, subjects first completed a brief evaluation of their own condition before being presented with the task, which included a pair of images distinguished by color and category. Once the sequence started, they were required to make distinctions based first on color, followed by category. If the color did not match the target (Non-Target, NT case), no processing was necessary. When only the color matched, a mental computation was performed (Shallow Target, ST case). If both the color and category matched the target (Deep Target, DT case), the subjects had to perform a related cognitive task and additional mental computations for evaluation. The results regarding the number of correct distinctions were saved at the end of each run. This cognitive task was performed in five runs per condition. The conditions were kept consistent throughout the trials to avoid confusion among the different task forms. This task was chosen for the cognitive analysis in this study because it involves memory retrieval by comparing a previously presented stimulus with the current stimulus, which is associated with cognitive workload [51]. In a visual stimulus paradigm with two degrees of cognitive processing, shallow processing is associated with a low level of cognition, and deep processing is associated with a high level of cognition [98].

A total of 600 trials, consisting of five runs, each lasting 1.25 s, were conducted, divided into 75% NT cases and 12.5% ST or DT cases, consistent with the proposed occurrence rate for positive targets [99]. A total of 120 stimuli, each comprising a pair of two images with the same color (red, green, blue, or magenta) were presented after a cue. Each image pair depicted different objects selected from the categories of animals, fruits, and mobility, with each category containing a total of ten objects.

### 2.3. Preprocessing of Emotional Data

EEG data were collected at a sampling rate of 250 Hz, and subsequently, a band-pass filter was applied in order to focus on critical EEG frequency bands within the range of 0.1 to 40 Hz with a band-pass sixth-order Butterworth filter. Moreover, a 50 Hz notch filter was applied for line noise cancellation. The max–min method eliminates artifactual epochs by examining their features and identifying whether they fall outside of the typical threshold range. For eye movement artifacts, defined as those exceeding a definite value (100 μV in our case) in amplitude, a threshold is established at a maximum difference of a definite value (150 μV in our case) between the highest and lowest amplitude values within a single epoch. If the maximum difference of the epoch in at least one channel exceeds the threshold, the epoch is excluded from the analysis. Artifacts arising as a result of eye blinks or facial movements in the two-channel EOG data were identified through raw data inspection and were removed using the ICA method with EEGLAB [100]. We first performed a detailed visual inspection of the raw EEG data with the aim of catching abnormal patterns caused by spikes, flat lines, or noise. Later, other high-frequency components and unwanted signals arising from artifacts, including line interference, were eliminated with frequency analysis. Another criterion for artifact identification is the abnormality in the duration or amplitude of a signal. Any signal exceeding a definite value not compatible with a typical EEG pattern was noted as an artifact. Experienced psychologists or clinicians were consulted to validate the findings during the inspection process. The Infomax algorithm for ICA was used within EEGLAB. ICA is a linear decomposition method that allows for the identification of irrelevant or undesired constituents of the signal, which can be easily identified as artifacts by visual inspection. While our preprocessing pipeline affected data rank, we used the PCA option to handle it, matching the data rank to avoid the ghosting issue [101]. After removing these artifacts, EEG data can be obtained in a more purified form. Epochs were obtained by using segments starting 200 ms before the stimulus was presented and ending 1000 ms later, giving each segment a total length of 1200 ms. Baseline correction was applied for the 200 ms preceding the presentation of the stimulus.

### 2.4. Preprocessing of Cognitive Data

Cognitive EEG data were collected at a sampling rate of 1 kHz and were later downsampled to 100 Hz. The EEG signals were re-referenced to the left (A2) and right (Ref) mastoid channels. Therefore, data were obtained for 62 scalp channels and one EOG channel (Fp1, Fp2, AF7, AF3, AF4, AF8, F9, F7, F5, F3, F1, Fz, F2, F4, F6, F8, F10, FT7, FC5, FC3, FC1, FCz, FC2, FC4, FC6, FT8, C5, C3, C1, Cz, C2, C4, C6, T8, TP7, CP5, CP3, CP1, CPz, CP2, CP4, CP6, TP8, P9, P7, P5, P3, P1, Pz, P2, P4, P6, P8, P10, PO7, PO3, POz, PO4, PO8, O1, Oz, O2, EOG). After extracting the EOG channel, a zero-phase, non-causal FIR filter with an order of 43, a 32 Hz band-passedge frequency, and a 36 Hz cutoff frequency (−6 dB) was applied [100]. Then, a 1 Hz FIR filter of order 300 was used, using least-squares error minimization and reverse digital filtering with zero-phase effect. For line noise cancellation, a 50 Hz notch filter was used. Channels with a variance of less than 0.5 μ$V^{2}$ in more than 10% of the trials were removed. Moreover, epochs containing muscle artifacts were also extracted, characterized by excessive variance in more than 20% of the channels. A max–min criterion was determined, and epochs with a difference of more than 150 μV between the maximum and minimum voltage in channels F9, F10, AF3, and AF4 were removed to eliminate eye-movement artifacts [102].

Independent Component Analysis (ICA) with artifactual component selection achieved using the Multiple Artifact Rejection Algorithm (MARA) [103] was applied to suppress artifacts, including smaller eye-movement artifacts, muscular artifacts, and loose electrodes. Moreover, visual inspection of each ICA component was conducted by considering the power spectral density and its topographic distribution.

The EEG data were segmented into epochs with a fixation duration of 500 ms before the stimulus was presented, followed by presentation of the stimulus for 1250 ms and a relaxation period of 750 ms. Baseline correction was applied for the 200 ms before the presentation of the stimulus in order to suppress background noise in each epoch.

### 2.5. ERP Analysis of Emotional Data

**Figure 4:**
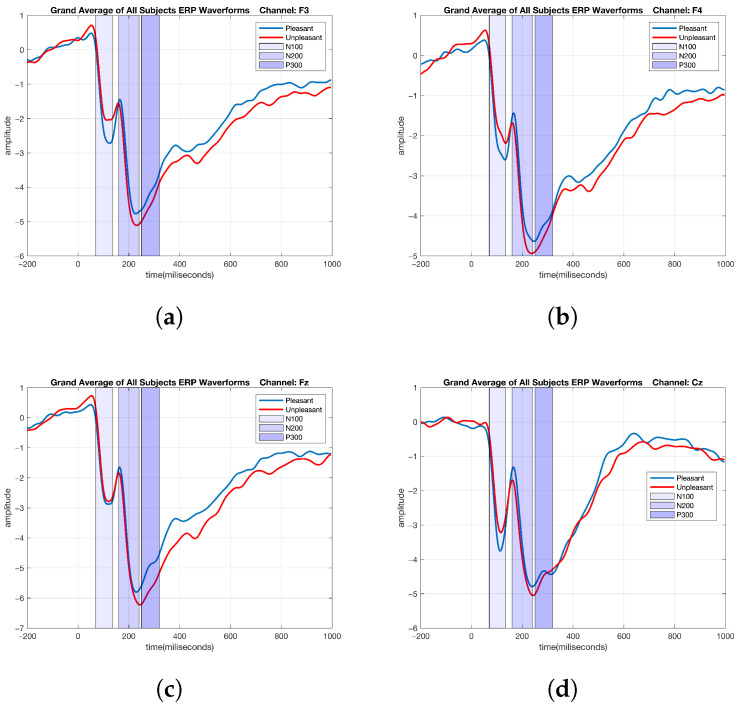
Emotional ERP waveforms based on the grand averages of all subjects: the F3, F4, Fz, and Cz channels. (**a**) F3 channel. (**b**) F4 channel. (**c**) Fz channel. (**d**) Cz channel.

After preprocessing, the remaining valid responses to each stimulus type, namely pleasant and unpleasant, were averaged for each subject. The grand average for each electrode was then calculated by averaging these values across subjects to obtain the ERPs. For each type of stimulus, the ERPs were computed by averaging the epochs that were time-locked to a specific event, which is a commonly used method in EEG data analysis [104]. Subsequently, epochs associated with pleasant and unpleasant stimuli were averaged separately across all subjects to determine where there were differences in the ERP responses when the subjects were exposed to images that elicited pleasant versus unpleasant emotional moods. The EEG channels and time intervals are represented in Figure 4 by the grand-average ERP waveforms evoked by pleasant and unpleasant stimuli.

Upon examining the ERP differences between the pleasant and unpleasant conditions across all of the channels, it is evident that the ERP amplitude for pleasant stimuli was greater than that for unpleasant stimuli during certain time intervals in various channels.

### 2.6. ERP Analysis of Cognitive Data

**Figure 5:**
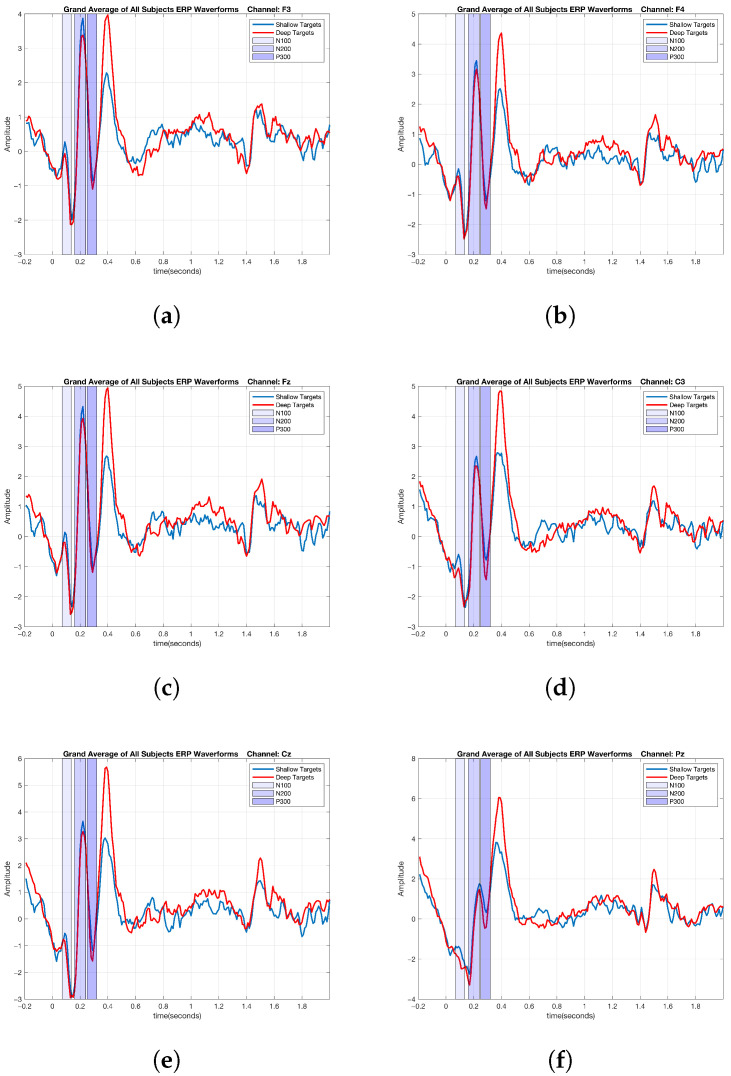
Cognitive ERP waveforms based on the grand averages of all subjects: the F3, F4, Fz, C3, Cz, and Pz channels. (**a**) F3 channel. (**b**) F4 channel. (**c**) Fz channel. (**d**) C3 channel. (**e**) Cz channel. (**f**) Pz channel.

After obtaining the epochs for the low-level and high-level cognitive tasks across all the subjects, ERP analysis was performed to determine whether there were differences in the results between the two tasks. The grand averages of ERPs were calculated by averaging across all of the trials and subjects for each condition. This approach aimed to reveal neurophysiological differences in the participants’ responses to the two different cognitive tasks. As the most significant differences between the two cognitive tasks were observed in the F3, F4, Fz, C3, Cz, and Pz channels, the ERPs obtained from these channels are presented here. Based on the grand averages of the ERPs across all of the subjects and trials for the two conditions, distinctions between the low-level and high-level cognitive tasks are evident in the N100, N200, and P300 ERP components, as shown in Figure 5.

### 2.7. Wavelet Coherence Analysis of Emotional Data

Wavelet coherence can be used to analyze the relationship between two signals by estimating their spectral patterns and is frequently used to analyze non-stationary signals based on continuous wavelet transform [105]. While coherence evaluates only the spectral components, without considering time information, wavelet coherence takes into account the coherence between two time-series signals within the time-frequency domain. This approach allows for the assessment of the degree of connectivity in multi-channel EEG signals and the spatial patterns of these connections. Consequently, this study investigated the potential of wavelet coherence as a feature extraction method for the analysis of EEG signals. In this way, the degree of connectivity among different multi-channel EEG signals and the spatial patterns arising from these connections can be obtained. Therefore, the aim of this section is to inspect the coherence among brain regions based on both the emotional and cognitive data.

The wavelet auto-spectrum of a signal (x(t)) is given as

(1) $$W_{xx}\left(t,f\right)=\int_{t-\delta /2}^{t+\delta /2}S_{x}\left(\tau ,f\right)\cdot S_{x}^{*}\left(\tau ,f\right)d\tau$$

where *t* and *f* are the time and frequency and * is the complex conjugation. δ is defined as the range of integration and is computed by $\delta =\frac{c}{f}$, where *c* is the number of integration cycles in the wavelet window.

The wavelet cross-spectrum between two signals (x(t) and y(t)) is similarly defined as

(2) $$W_{xy}\left(t,f\right)=\int_{t-\delta /2}^{t+\delta /2}S_{x}\left(\tau ,f\right)\cdot S_{y}^{*}\left(\tau ,f\right)d\tau$$

Wavelet coherence is the normalized wavelet cross-spectrum and is given as

(3) $$WCoh_{xy}\left(t,f\right)=\frac{|W_{xy}\left(t,f\right)|}{\sqrt{W_{xx}\left(t,f\right).W_{yy}\left(t,f\right)}}$$

In this study, Wavelet coherence was computed in MATLAB R2024a with the Signal Processing Toolbox following the algorithm proposed in [106]. The prototype function used for wavelet analysis was a Morlet wavelet. The minimum and maximum scales were selected such that lowest frequency was limited by the signal length and the wavelet’s time-frequency extent and highest frequency were limited by the Nyquist frequency. The number of scales was floor$(\log_{2}\left(N\right))-1$, where *N* is the number of samples in the input signal.

**Figure 6:**
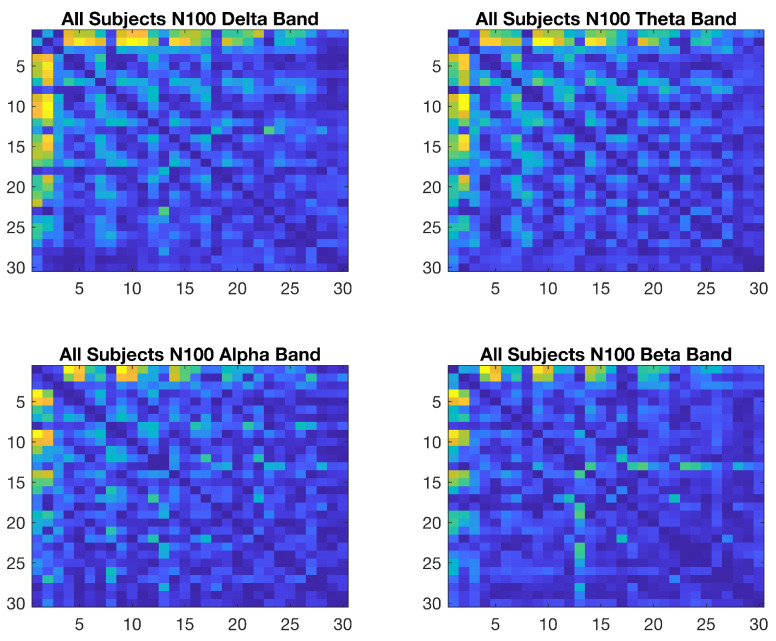
Emotional N100 connectivity maps of difference for the delta, theta, alpha, and beta frequency bands.

The wavelet coherence values for the N100, N200, and P300 time intervals among all of the channels were calculated for the delta, theta, alpha, and beta frequency bands and were calculated separately for each subject and for each trial. As three distinct ERP components, the N100 interval, relating to sensory activation; the N200 interval, originating from the anterior cingulate and fronto-central regions; and the P300 interval, indicating a measure of parietal-frontal connectivity, are all assumed to represent a hierarchical model used in understanding the mechanism of emotion and cognition within milliseconds [107]. Therefore, these components were employed in wavelet coherence analysis with the expectation that they would help discriminate the emotional states, as well as the cognitive state, in an accurate way. Before investigating the subjects individually, averages of all of the subjects’ wavelet coherence values over all the trials were obtained. The differences between the pleasant and unpleasant conditions are shown in Figure 6. Lighter hues indicate larger differences, whereas darker hues denote smaller ones in the figure.

Connectivity maps were also obtained for the N200 and P300 ERP components and showed similar patterns, as illustrated in Figure 6. EEG channels FP1, FP2, F3, Fz, F4, FC3, FCz, FC4, F7, F8, FT7, FT8, C3, Cz, C4, CP3, CPz, CP4, T7, T8, P3, Pz, P4, TP7, TP8, P7, P8, O1, Oz, and O2 are numbered from 1 to 30 on both axes.

**Figure 7:**
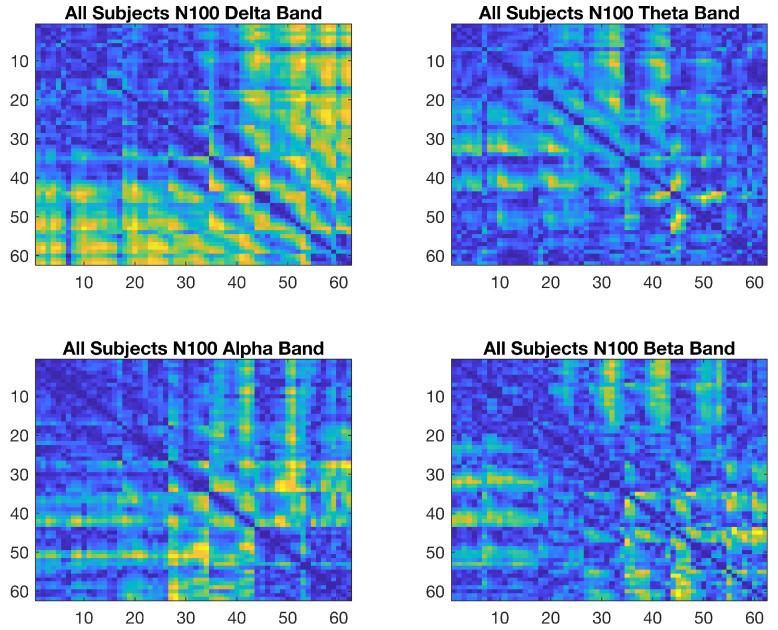
Cognitive N100 connectivity maps of difference for the delta, theta, alpha, and beta frequency bands.

The same procedure used for processing the emotional data was also applied to the cognitive data. The wavelet coherence values of the N100, N200, and P300 ERP segments for low and high cognitive states were calculated for 62 channels in the delta, theta, alpha, and beta frequency bands. Maps showing the wavelet coherence value differences between the low and high cognition states for the N100 ERP component are shown in Figure 7.

Connectivity maps were also obtained for the N200 and P300 ERP components. EEG channels FP1, FP2, F3, Fz, F4, FC3, FCz, FC4, F7, F8, FT7, FT8, C3, Cz, C4, CP3, CPz, CP4, T7, T8, P3, Pz, P4, TP7, TP8, P7, P8, O1, Oz, and O2 are numbered from 1 to 62 on both axes.

## 3. Results

### 3.1. Graph-Theoretic-Based Analysis of Emotional Data

Global and local graph metrics such as energy, efficiency, strength, transitivity, characteristic path lengths, and clustering coefficients were computed for the pleasant and unpleasant conditions using the Brain Connectivity Toolbox [108] based on the connectivity maps obtained for each ERP component and for each frequency band.

Once the global and local graph metrics from the original graphs had been calculated, the same computations were repeated for 100 surrogate graphs. Threshold values are typically determined heuristically [108], as there is no definitive criterion for their selection [109]. While generating the surrogate networks, various threshold values within the 0.1–0.3 range were applied in incremental steps of 0.1. These sparsity levels were selected because the brain network usually falls within this range [110]. After the graph metrics for the original and surrogate graphs had been calculated, z-score normalization was applied to normalize the data. The mean value of each surrogate parameter was subtracted from the original value and divided by the standard deviation.

**Figure 8:**
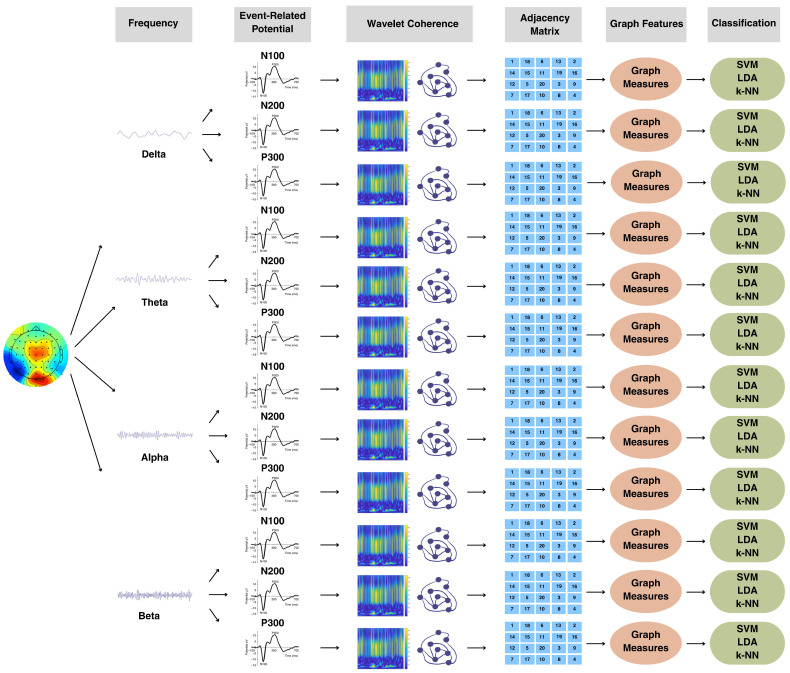
Pipeline for classification based on graph metrics.

The strength and clustering coefficient, as two local measures for each channel, and the energy, efficiency, transitivity, and characteristic path length, as global measures, were chosen, resulting in a set of 2 × 30 + 4 = 64 features. The most discriminant features were selected by applying two different selection methods. A *t*-test was performed for each feature over two classes. Alternatively, the ReliefF algorithm [36,111,112] was applied. The top 20 most discriminative features identified by ReliefF were then used in the classification stage. In addition, the minimum-redundancy, maximum-relevance (MRMR) feature selection technique was applied, although it does not perform as well as the ReliefF and *t*-test methods. The K-NN, LDA, and SVM classification methods were applied separately for the delta, theta, alpha, and beta bands. The classification process was implemented using 10-fold cross-validation repeated 100 times. We performed within-subject cross-validation, splitting each participant’s trials into training and test folds. In the SVM method, a radial basis function (RBF) kernel was used, with the kernel scale fixed at one. Bayesian optimization was selected in hyperparameter optimization. As for the LDA method, Fisher’s LDA was set as the discriminant type, and regularization parameters were set to 0. No hyperparameter tuning parameter was used. In the K-NN method, Euclidean distance was chosen as the distance metric, with the number of neighbors set to one. The distance weight was set as equal, and no hyperparameter tuning parameter was selected. Additionally, the features extracted from each ERP component and their combinations were used separately for classification within each frequency band, that is, for delta-band classification, features corresponding to N100, N200, and P300, as well as their combinations—N100 and N200, N100 and P300, N200 and P300, and N100, N200, and P300—were used sequentially. The analysis pipeline is shown in Figure 8.

An extensive analysis was performed for each frequency band, and it was observed that the classification results for the theta, alpha, and beta bands did not exceed an accuracy of 65%. This observation was consistent for each individual subject in both datasets. Therefore, our subsequent analyses were focused on the delta band, which yielded the highest discriminatory performance. Among the various feature combinations used for classification, the best accuracy was achieved using features from the delta band and the combined N100, N200, and P300 ERP components selected using the *t*-test and ReliefF methods, along with the SVM classifier. The SVM classification results for each subject are presented in Table 1. The classification process was performed using 10-fold cross-validation repeated 100 times, and the reported results represent the average over these 100 iterations. The classification results were analyzed using the *t*-test method, and an average classification performance of 89.9% was observed across the subjects, with the maximum classification performance of 96.1% achieved for Subject 5. As for the ReliefF method, an average classification performance of 91.8% was observed across the subjects, with the maximum classification performance of 96.5% achieved for Subject 5.

### 3.2. Graph-Theoretic-Based Analysis of Cognitive Data

The same preprocessing and feature extraction methods employed for the classification of the emotional data, as described in the Section 3.1, were also used for the cognitive data.

The top 10 most discriminative features identified by ReliefF were then used in the classification stage. The K-NN, LDA, and SVM classification methods were utilized for the delta, theta, alpha, and beta bands to discriminate low and high cognitive states. Moreover, the features extracted from each ERP component and their combination were used in the classification for each band, just as in the case of the emotional data. The classification process was implemented using 10-fold cross-validation repeated 100 times, and the reported results represent the average over these 100 iterations.

The highest classification rate was obtained with the SVM method using the features from the delta band belonging to the N100, N200, and P300 ERP components in combination. The SVM classification results obtained for each subject using the two different feature selection methods are presented in Table 2. According to the analysis of the classification results using the *t*-test method, an average classification performance of 83.5% was observed across the subjects, with the maximum classification performance of 93.6% achieved for Subject 5. As for the ReliefF method, an average classification performance of 88.5% was observed across the subjects, with the maximum classification performance of 92.2% achieved for Subject 5.

## 4. Discussion

### 4.1. Comparison with Related Work

This study investigated local and global graph-theoretic features obtained from wavelet coherence-based connectivity maps for different ERP components.

Both emotional and cognitive data were analyzed in order to validate the proposed method. Graph-theoretic metrics obtained from wavelet coherence values were used to classify the data collected for the two conditions. Various EEG studies on graph-based classification have been reported in the literature, with many employing connectivity maps based on PLVs [71,77,78]. Some studies have used wavelet coherence values to compute graph-theoretic measures, but they did not focus on individual ERP components [23,113]. To the best of our knowledge, this is the first study investigating graph-theoretic metrics in different frequency bands by including wavelet coherence values obtained from various ERP components.

It was found that some channels—especially F3, F4, C3, and Cz more clearly discriminated the two conditions in each dataset. This is consistent with other emotionally and cognitively based studies that have found the F3, F4, and Fz channels to be particularly notable [114,115]. According to the results reported in this study, it can be concluded that selecting a combination of different ERP components for feature selection allows for greater success in the classification of different cases in both types of data. The ST-DT discrimination performance was enhanced to 88.5% with the proposed method, compared to the performance of around 72% reported in [102] that was obtained using the same data. Discrimination between the two cases for both data types was achieved with a high success rate of over 90% in the delta band. This finding aligns with those of another study [116], which highlighted that lower-frequency waves are often associated with affective processes. Furthermore, it has been shown that delta-band activity is associated with long-range cortico-cortical functional connectivity, which is crucial for comprehending the organization and communication of large-scale brain networks [117]. Based on our evaluation of the classification performance in the delta band, it can be concluded that delta-band connectivity patterns exhibit considerable modulation during emotional and cognitive processing. Classification based on the emotional data yielded more promising results when multiple time intervals were used, an observation that is consistent with the results of another study that selected several ERP components in its classification analysis [49]. This emphasizes the importance of using wavelet coherence values for the N100, N200, and P300 ERP components in combination when classifying emotional data. Our results are in accordance with the findings of a study that demonstrated the importance of using ERP components other than P300 in the recognition of facial emotions [47]. The integration of ERP components into connectivity analysis offers insight into neural activity over time in different brain regions during the performance of tasks [118]. This widens the scope of neural activity processing during emotional and cognitive tasks. In another study [16] that used the DEAP dataset and applied mutual information to obtain graph-based features, in addition to statistical features from peripheral physiological signals, classification rates of 88.3% and 90.8% were obtained for valence and arousal, respectively. Another study [78] used PLV-based graphs to obtain connectivity metrics, and a classification of 84.4% based on the SEED dataset was achieved. The same study obtained average accuracies of 73.3%, 77.0%, and 79.2% for valence, arousal, and dominance classifications, respectively, based on the DEAP database. In addition to emotional and cognitive data analysis, motor imagery data have also been analyzed using graph metrics [109], although a classification performance of 87% was achieved, which is inferior to this study’s results. The selection of different threshold values for generating surrogate graphs did not yield any large differences. As for feature selection, the ReliefF method demonstrated better classification performance compared to the *t*-test method on both datasets.

### 4.2. Interpretation of Key Findings

Among all graph-theoretic metrics, the clustering coefficient parameter was found to be the most discriminatory feature between the two conditions in both datasets. This finding shows a condition-specific reorganization of local network segregation. It suggests that the balance between short-range feedback connections and long-range integration varies systematically between the two different classes [108]. A larger clustering coefficient in one group indicates a denser presence of triangular motifs among neighboring nodes, which aligns with more closely connected, functionally specialized subnetworks designed for quick, context-dependent information exchange [119]. This arrangement is often viewed as enhancing fault tolerance and improving processing efficiency within local circuits. Condition-dependent increases in clustering are typically linked to heightened attentional demands or emotional arousal [120]. In contrast, lower clustering is often associated with states that promote distributed processing or globally synchronous dynamics, such as relaxed wakefulness or specific sleep stages. It is a general fact that cognitive tasks are usually more demanding than emotional tasks, despite bringing about a similar complexity change in the structural features of the data. Part of the reduction in cognitive classification over emotion may be attributed to its relative complexity and demandedness. Another possible reason is that cognitive tasks generate overlapping neural activations, causing less distinct patterns compared to emotional tasks.

Delta waves are produced by extensive, reciprocally connected cortico-subcortical loops, and their long cycle length accommodates axonal conduction delays, making them well-suited for coordinating activity across distant regions of the brain [121,122,123]. Studies indicate that as the spatial extent of a network increases, the preferred oscillatory frequency decreases; therefore, large-scale interactions are typically observed in the delta/theta range [124]. In one study [125], it was shown that the effective down-regulation of affect enhances delta-band synchrony in prefrontal–limbic pathways and various other emotion networks. The same circuits facilitate cognitive control over affect, providing a plausible explanation for why delta connectivity encompasses joint information about both areas in our dataset. Delta oscillations exhibit a higher signal-to-noise ratio in the scalp [126] and are less affected by muscle artifacts compared to beta–gamma activity. Their slower time scale allows for more reliable estimates of phase-based connectivity (such as wavelet coherence) over the duration typically utilized in emotion–cognition paradigms. These statistical advantages result in the generation of cleaner adjacency matrices, which leads to more distinct graph-theoretic metrics. Consistent with our findings about clustering coefficients for emotional data, a study [119] reported on clustering coefficient discrimination between different emotions considering connectivity in the delta band. As for cognitive data analysis, another study [120] reported on clustering coefficient discrimination between two different cognitively loaded tasks, in line with the results presented in our study. The observation that delta-band connectivity prevails in classification indicates that adaptive brain–computer interfaces should emphasize slow-frequency network features, particularly when user states are influenced by a combination of emotional and cognitive factors.

### 4.3. Methodological Implications

After evaluating the classification results across all of the subjects, the results of this study confirm that changes in cognitive workload and pleasant versus unpleasant emotional moods can be classified with high success rates using the SVM algorithm after deriving graph-theoretic metrics based on wavelet coherence values in certain frequency bands of ERP components. The method proposed in this study could potentially be applied to different types of EEG data.

## 5. Conclusions

### 5.1. Future Work

BCI technology remains a challenging and futuristic research area that is still in its earliest stages of exploration. Graph-theoretic metrics based on wavelet coherence yield promising results in the discrimination of emotional and cognitive states. Therefore, future BCI studies that focus on this research direction are warranted. In addition, the application of the method proposed in this study can be extended to other types of data, such as motor imagery or inner reading. The results obtained in this study imply that the proposed method is a viable approach to understanding and investigating emotional and cognitive states under different conditions using EEG data. A promising direction for subsequent research is the development of integrated neurofeedback protocols that concurrently modulate emotional and cognitive processes. Rigorous design and evaluation of such designs could be beneficial in geriatric and educational methods.

### 5.2. Limitations

It is definitely an important aspect in such a study to increase the number of subjects to improve the statistical power. However, in this work, we focused on a classification method based on individual subjects whose datasets consisted of more than 500 trials each for training and testing. The accuracy results we for through 13 (15) subjects did not vary by a margin of more than 7% percent, as presented in Table 1 and Table 2. Therefore, we were able to achieve a certain level of consistency across subjects in terms of classification performance, even though the subjects do represent a larger group also encountered in similar studies [127,128,129,130]. A principal limitation of current BCI systems is their requirement for subject-specific calibration, and optimal performance can be achieved only by training the system individually for each user. In addition, EEG data were collected from each subject in one session only. The validity of the results could be improved by using data collected over multiple sessions to account for variability due to biorhythms. To overcome these limitations, multiple sessions of individual data recording would enhance the training performance of the BCI systems. Furthermore, emotional responses may vary as a result of sociological and cultural differences [16]. To mitigate the effects of such variability, emotional data could be collected from people raised in different cultural settings. Finally, the basis for emotional and cognitive data analysis could be extended to other types of physiological signals [131], such as electrocardiography, electro-dermal activity, blood pressure, and respiration.

## Figures and Tables

**Table 1:** The SVM classification results for each subject obtained based on the N100, N200, and P300 delta graph features for the emotional data.

Subject	*t*-Test Feature Selection (%)				ReliefF Feature Selection (%)			
	**Accuracy**	**Sensitivity**	**Specificity**	**F1 Score**	**Accuracy**	**Sensitivity**	**Specificity**	**F1 Score**
Subject-1	87.5	89.0	86.1	87.9	91.9	92.4	90.5	91.6
Subject-2	87.3	89.1	85.4	87.6	90.7	90.6	88.6	89.8
Subject-3	92.4	95.1	89.6	92.6	93.2	94.2	91.4	92.9
Subject-4	84.6	85.2	84.3	84.9	89.8	91.9	88.6	90.4
Subject-5	96.1	96.7	95.5	96.2	96.5	97.7	94.0	96.0
Subject-6	91.0	92.8	89.1	91.2	91.1	89.7	90.2	90.0
Subject-7	90.7	92.2	89.4	90.9	93.0	93.1	91.6	92.4
Subject-8	88.3	83.5	93.4	87.9	90.6	88.9	93.4	91.0
Subject-9	91.1	91.9	90.4	91.2	91.5	92.7	90.6	91.8
Subject-10	91.2	90.1	92.4	91.2	89.5	88.4	90.7	89.5
Subject-11	84.3	81.5	86.8	83.8	90.4	89.2	92.9	90.9
Subject-12	93.0	91.5	94.6	92.9	95.7	94.6	96.0	95.3
Subject-13	91.6	95.8	87.2	91.8	92.3	95.0	90.2	92.8
Average	89.9	90.3	89.6	90.0	91.8	92.2	91.4	91.9

**Table 2:** The SVM classification results for each subject, obtained based on the N100, N200, and P300 delta graph features for the cognitive data.

Subject	*t*-Test Feature Selection (%)				ReliefF Feature Selection (%)			
	**Accuracy**	**Sensitivity**	**Specificity**	**F1 Score**	**Accuracy**	**Sensitivity**	**Specificity**	**F1 Score**
Subject-1	82.4	81.7	83.3	82.6	90.2	91.0	89.2	90.3
Subject-2	87.4	89.5	85.5	87.9	85.8	88.0	83.6	86.3
Subject-3	90.4	92.7	88.5	90.9	90.8	91.9	89.6	91.0
Subject-4	89.9	89.1	90.7	89.9	91.3	90.2	92.8	91.5
Subject-5	93.6	95.0	92.1	93.7	92.2	93.3	90.9	92.3
Subject-6	88.6	88.6	88.0	88.5	88.0	85.8	90.3	87.9
Subject-7	91.6	89.9	93.4	91.6	85.8	85.5	86.2	86.0
Subject-8	69.2	71.4	70.2	70.9	88.0	88.9	87.2	88.4
Subject-9	80.0	82.3	83.1	80.3	87.7	89.1	86.3	88.1
Subject-10	75.6	77.0	78.1	76.2	91.1	91.2	91.0	91.3
Subject-11	78.8	80.2	81.0	79.8	89.0	88.7	89.7	89.3
Subject-12	75.2	77.4	76.5	76.1	86.3	83.4	89.3	86.2
Subject-13	73.1	75.2	74.7	74.1	87.2	85.4	89.1	89.6
Subject-14	87.9	86.3	89.2	87.7	89.3	89.8	89.1	89.6
Subject-15	88.4	82.5	94.4	87.8	85.2	85.2	85.2	85.3
Average	83.5	83.9	84.6	83.9	88.5	88.5	88.6	88.7

## Data Availability

The emotional data presented in this study are available upon request from the corresponding author due to privacy reasons. Cognitive data were obtained from the DepositOnce repository of Technische Universität Berlin (https://depositonce.tu-berlin.de/handle/11303/6747, accessed on 17 April 2020). More details regarding the data are published in [96].

## Abbreviations

The following abbreviations are used in this manuscript:

AgCl	Silver chloride
BCI	Brain–computer interface
DEAP	Database for Emotion Analysis using Physiological Signals
DT	Deep target
ECG	Electrocardiography
EEG	Electroencephalography
EMG	Electromyography
EOG	Electro-oculography
ERP	Event-related potential
FIR	Finite impulse response
fMRI	Functional magnetic resonance imaging
ICA	Independent component analysis
K-NN	K-nearest neighbor
LDA	Linear discriminant analysis
MARA	Multiple artifact rejection algorithm
MEG	Magnetoencephalography
MRMR	Minimum redundancy, maximum relevance
NT	Non-target
N100	Negative 100
N200	Negative 200
PLV	Phase-locking value
P300	Positive 300
RBF	Radial basis function
SEED	The SJTU Emotion EEG Dataset (SEED)
ST	Shallow target
SVM	Support vector machine

## References

[1] Maddirala A.K., Veluvolu K.C. (2021). Eye-blink artifact removal from single channel EEG with k-means and SSA. Sci. Rep..

[2] Fragopanagos N., Taylor J.G. (2005). Emotion recognition in human–computer interaction. Neural Netw..

[3] Spezialetti M., Placidi G., Rossi S. (2020). Emotion recognition for human-robot interaction: Recent advances and future perspectives. Front. Robot. AI.

[4] Li J., Zhang Z., He H. (2016). Implementation of EEG emotion recognition system based on hierarchical convolutional neural networks. Proceedings of the Advances in Brain Inspired Cognitive Systems: 8th International Conference, BICS 2016.

[5] Kothe C.A., Makeig S. (2011). Estimation of task workload from EEG data: New and current tools and perspectives. Proceedings of the 2011 Annual International Conference of the IEEE Engineering in Medicine and Biology Society.

[6] Yokota Y., Naruse Y. (2015). Phase coherence of auditory steady-state response reflects the amount of cognitive workload in a modified N-back task. Neurosci. Res..

[7] Lin C.J., Hsieh M.H. (2009). Classification of mental task from EEG data using neural networks based on particle swarm optimization. Neurocomputing.

[8] Shi L.C., Lu B.L. (2013). EEG-based vigilance estimation using extreme learning machines. Neurocomputing.

[9] Liu Y.H., Wu C.T., Cheng W.T., Hsiao Y.T., Chen P.M., Teng J.T. (2014). Emotion recognition from single-trial EEG based on kernel Fisher’s emotion pattern and imbalanced quasiconformal kernel support vector machine. Sensors.

[10] Liu Y., Sourina O., Nguyen M.K. (2011). Real-time EEG-based emotion recognition and its applications. Transactions on Computational Science XII: Special Issue on Cyberworlds.

[11] Basar M.D., Duru A.D., Akan A. (2020). Emotional state detection based on common spatial patterns of EEG. Signal Image Video Process..

[12] Ma W., Zheng Y., Li T., Li Z., Li Y., Wang L. (2024). A comprehensive review of deep learning in EEG-based emotion recognition: Classifications, trends, and practical implications. PeerJ Comput. Sci..

[13] Balli T., Deniz S.M., Cebeci B., Erbey M., Duru A.D., Demiralp T. (2013). Emotion recognition based on spatially smooth spectral features of the EEG. Proceedings of the 2013 6th International IEEE/EMBS Conference on Neural Engineering (NER).

[14] Dong S., Reder L.M., Yao Y., Liu Y., Chen F. (2015). Individual differences in working memory capacity are reflected in different ERP and EEG patterns to task difficulty. Brain Res..

[15] Thejaswini S., Kumar K.R., Vijayendra A., Shyam R., Anchan P.D., Gowda E. (2017). An algorithm to detect emotion states and stress levels using EEG signals. Int. J. Latest Res. Eng. Technol. (IJLRET).

[16] Xefteris V.R., Tsanousa A., Georgakopoulou N., Diplaris S., Vrochidis S., Kompatsiaris I. (2022). Graph theoretical analysis of eeg functional connectivity patterns and fusion with physiological signals for emotion recognition. Sensors.

[17] Kim M.K., Kim M., Oh E., Kim S.P. (2013). A review on the computational methods for emotional state estimation from the human EEG. Comput. Math. Methods Med..

[18] Davidson R.J., Fox N.A. (1982). Asymmetrical brain activity discriminates between positive and negative affective stimuli in human infants. Science.

[19] Pfurtscheller G., Leeb R., Keinrath C., Friedman D., Neuper C., Guger C., Slater M. (2006). Walking from thought. Brain Res..

[20] Elfenbein H.A., Ambady N. (2002). Predicting workplace outcomes from the ability to eavesdrop on feelings. J. Appl. Psychol..

[21] Cecchetto C., Korb S., Rumiati R.I., Aiello M. (2018). Emotional reactions in moral decision-making are influenced by empathy and alexithymia. Soc. Neurosci..

[22] Preuss N., Brändle L.S., Hager O.M., Haynes M., Fischbacher U., Hasler G. (2016). Inconsistency and social decision making in patients with Borderline Personality Disorder. Psychiatry Res..

[23] Si Y., Li F., Duan K., Tao Q., Li C., Cao Z., Zhang Y., Biswal B., Li P., Yao D. (2020). Predicting individual decision-making responses based on single-trial EEG. NeuroImage.

[24] Chikhi S., Matton N., Blanchet S. (2022). EEG power spectral measures of cognitive workload: A meta-analysis. Psychophysiology.

[25] Kahneman D. (1973). Attention and Effort.

[26] Torres E.P., Torres E.A., Hernández-Álvarez M., Yoo S.G. (2020). EEG-based BCI emotion recognition: A survey. Sensors.

[27] Makeig S., Kothe C., Mullen T., Bigdely-Shamlo N., Zhang Z., Kreutz-Delgado K. (2012). Evolving signal processing for brain–computer interfaces. Proc. IEEE.

[28] Aricò P., Borghini G., Di Flumeri G., Colosimo A., Pozzi S., Babiloni F. (2016). A passive brain–computer interface application for the mental workload assessment on professional air traffic controllers during realistic air traffic control tasks. Prog. Brain Res..

[29] Di Flumeri G., Borghini G., Aricò P., Colosimo A., Pozzi S., Bonelli S., Golfetti A., Kong W., Babiloni F. (2015). On the use of cognitive neurometric indexes in aeronautic and air traffic management environments. Proceedings of the Symbiotic Interaction: 4th International Workshop, Symbiotic 2015.

[30] Brouwer A.M., van de Water L., Hogervorst M., Kraaij W., Schraagen J.M., Hogenelst K. (2018). Monitoring mental state during real life office work. Proceedings of the Symbiotic Interaction: 6th International Workshop, Symbiotic 2017.

[31] Venthur B., Blankertz B., Gugler M.F., Curio G. (2010). Novel applications of BCI technology: Psychophysiological optimization of working conditions in industry. Proceedings of the 2010 IEEE International Conference on Systems, Man and Cybernetics.

[32] Maimon N.B., Molcho L., Intrator N., Lamy D. (2020). Single-channel EEG features during n-back task correlate with working memory load. arXiv.

[33] Pesonen M., Hämäläinen H., Krause C.M. (2007). Brain oscillatory 4–30 Hz responses during a visual n-back memory task with varying memory load. Brain Res..

[34] Wang S., Gwizdka J., Chaovalitwongse W.A. (2015). Using wireless EEG signals to assess memory workload in the *n*-back task. IEEE Trans. -Hum.-Mach. Syst..

[35] Gaurav G., Anand R.S., Kumar V. (2021). EEG based cognitive task classification using multifractal detrended fluctuation analysis. Cogn. Neurodynamics.

[36] Joseph A.F.A., Govindaraju C. (2021). Minimizing electrodes for effective brain computer interface. Biomed. Signal Process. Control.

[37] Magosso E., De Crescenzio F., Ricci G., Piastra S., Ursino M. (2019). EEG alpha power is modulated by attentional changes during cognitive tasks and virtual reality immersion. Comput. Intell. Neurosci..

[38] Zhang Z.T., Argın S.K., Bilen M.B., Urgun D., Deniz S.M., Liu Y., Hassib M. (2024). Measuring the effect of mental workload and explanations on appropriate AI reliance using EEG. Behav. Inf. Technol..

[39] Bakhshali M.A., Ebrahimi-Moghadam A., Khademi M., Moghimi S. (2019). Coherence-based correntropy spectral density: A novel coherence measure for functional connectivity of EEG signals. Measurement.

[40] Demiralp T., Başar E. (1992). Theta rhythmicities following expected visual and auditory targets. Int. J. Psychophysiol..

[41] Demiralp T., Ademoglu A., Comerchero M., Polich J. (2001). Wavelet analysis of P3a and P3b. Brain Topogr..

[42] Başar E., Schürmann M., Demiralp T., Başar-Eroglu C., Ademoglu A. (2001). Event-related oscillations are ‘real brain responses’—Wavelet analysis and new strategies. Int. J. Psychophysiol..

[43] Blankertz B., Lemm S., Treder M., Haufe S., Müller K.R. (2011). Single-trial analysis and classification of ERP components—A tutorial. NeuroImage.

[44] Batty M., Taylor M.J. (2003). Early processing of the six basic facial emotional expressions. Cogn. Brain Res..

[45] Hofmann M.J., Kuchinke L., Tamm S., Võ M.L., Jacobs A.M. (2009). Affective processing within 1/10th of a second: High arousal is necessary for early facilitative processing of negative but not positive words. Cogn. Affect. Behav. Neurosci..

[46] Kissler J., Herbert C. (2013). Emotion, Etmnooi, or Emitoon?–Faster lexical access to emotional than to neutral words during reading. Biol. Psychol..

[47] Utama N.P., Takemoto A., Nakamura K., Koike Y. (2009). Single-trial EEG data to classify type and intensity of facial emotion from P100 and N170. Proceedings of the 2009 International Joint Conference on Neural Networks.

[48] Wang C., Xiong S., Hu X., Yao L., Zhang J. (2012). Combining features from ERP components in single-trial EEG for discriminating four-category visual objects. J. Neural Eng..

[49] Qin Y., Zhan Y., Wang C., Zhang J., Yao L., Guo X., Wu X., Hu B. (2016). Classifying four-category visual objects using multiple ERP components in single-trial ERP. Cogn. Neurodynamics.

[50] Wiens S., Sand A., Olofsson J.K. (2011). Nonemotional features suppress early and enhance late emotional electrocortical responses to negative pictures. Biol. Psychol..

[51] Kirchner W.K. (1958). Age differences in short-term retention of rapidly changing information. J. Exp. Psychol..

[52] Basar E., Demiralp T., Schürmann M., Basar-Eroglu C., Ademoglu A. (1999). Oscillatory brain dynamics, wavelet analysis, and cognition. Brain Lang..

[53] Demiralp T., Yordanova J., Kolev V., Ademoglu A., Devrim M., Samar V.J. (1999). Time–frequency analysis of single-sweep event-related potentials by means of fast wavelet transform. Brain Lang..

[54] Erdogdu E., Kurt E., Duru A.D., Uslu A., Başar-Eroğlu C., Demiralp T. (2019). Measurement of cognitive dynamics during video watching through event-related potentials (ERPs) and oscillations (EROs). Cogn. Neurodynamics.

[55] Straube S., Fahle M. (2010). The electrophysiological correlate of saliency: Evidence from a figure-detection task. Brain Res..

[56] Müller-Putz G.R., Riedl R., C Wriessnegger S. (2015). Electroencephalography (EEG) as a research tool in the information systems discipline: Foundations, measurement, and applications. Commun. Assoc. Inf. Syst..

[57] Demiralp T., Ademoglu A. (2001). Decomposition of event-related brain potentials into multiple functional components using wavelet transform. Clin. Electroencephalogr..

[58] Ademoglu A., Demiralp T., Yordanova J., Kolev V., Devrim M. (1998). Decomposition of event-related brain potentials into multicomponents using wavelet transform. Appl. Signal Process..

[59] Ademoglu A., Micheli-Tzanakou E., Istefanopoulos Y. (1997). Analysis of pattern reversal visual evoked potentials (PRVEPs) by spline wavelets. IEEE Trans. Biomed. Eng..

[60] Li M., Lu B.L. (2009). Emotion classification based on gamma-band EEG. Proceedings of the 2009 Annual International Conference of the IEEE Engineering in Medicine and Biology Society.

[61] Hadjidimitriou S.K., Hadjileontiadis L.J. (2013). EEG-based classification of music appraisal responses using time-frequency analysis and familiarity ratings. IEEE Trans. Affect. Comput..

[62] Zheng W.L., Lu B.L. (2015). Investigating critical frequency bands and channels for EEG-based emotion recognition with deep neural networks. IEEE Trans. Auton. Ment. Dev..

[63] Shi L.C., Jiao Y.Y., Lu B.L. (2013). Differential entropy feature for EEG-based vigilance estimation. Proceedings of the 2013 35th Annual International Conference of the IEEE Engineering in Medicine and Biology Society (EMBC).

[64] Tang C., Wang D., Tan A.H., Miao C. (2017). EEG-based emotion recognition via fast and robust feature smoothing. Proceedings of the Brain Informatics: International Conference, BI 2017.

[65] Akin M. (2002). Comparison of wavelet transform and FFT methods in the analysis of EEG signals. J. Med. Syst..

[66] Zhong P., Wang D., Miao C. (2020). EEG-based emotion recognition using regularized graph neural networks. IEEE Trans. Affect. Comput..

[67] Wu X., Zheng W.L., Lu B.L. (2019). Identifying functional brain connectivity patterns for EEG-based emotion recognition. Proceedings of the 2019 9th International IEEE/EMBS Conference on Neural Engineering (NER).

[68] Bullmore E., Sporns O. (2009). Complex brain networks: Graph theoretical analysis of structural and functional systems. Nat. Rev. Neurosci..

[69] Fingelkurts A.A., Fingelkurts A.A., Kähkönen S. (2005). Functional connectivity in the brain—Is it an elusive concept?. Neurosci. Biobehav. Rev..

[70] Greenblatt R.E., Pflieger M., Ossadtchi A. (2012). Connectivity measures applied to human brain electrophysiological data. J. Neurosci. Methods.

[71] Li P., Liu H., Si Y., Li C., Li F., Zhu X., Huang X., Zeng Y., Yao D., Zhang Y. (2019). EEG based emotion recognition by combining functional connectivity network and local activations. IEEE Trans. Biomed. Eng..

[72] Fallani F.D.V., Costa L.d.F., Rodriguez F.A., Astolfi L., Vecchiato G., Toppi J., Borghini G., Cincotti F., Mattia D., Salinari S. (2010). A graph-theoretical approach in brain functional networks. Possible implications in EEG studies. Proceedings of the Nonlinear Biomedical Physics.

[73] Stam C.J., Reijneveld J.C. (2007). Graph theoretical analysis of complex networks in the brain. Nonlinear Biomed. Phys..

[74] Ismail L.E., Karwowski W. (2020). A graph theory-based modeling of functional brain connectivity based on EEG: A systematic review in the context of neuroergonomics. IEEE Access.

[75] Ménoret M., Farrugia N., Pasdeloup B., Gripon V. (2017). Evaluating graph signal processing for neuroimaging through classification and dimensionality reduction. Proceedings of the 2017 IEEE Global Conference on Signal and Information Processing (GlobalSIP).

[76] Van Den Heuvel M.P., Pol H.E.H. (2010). Exploring the brain network: A review on resting-state fMRI functional connectivity. Eur. Neuropsychopharmacol..

[77] Gonuguntla V., Wang Y., Veluvolu K.C. (2013). Phase synchrony in subject-specific reactive band of EEG for classification of motor imagery tasks. Proceedings of the 2013 35th Annual International Conference of the IEEE Engineering in Medicine and Biology Society (EMBC).

[78] Wang Z., Tong Y., Heng X. (2019). Phase-locking value based graph convolutional neural networks for emotion recognition. IEEE Access.

[79] Chiarion G., Sparacino L., Antonacci Y., Faes L., Mesin L. (2023). Connectivity analysis in EEG data: A tutorial review of the state of the art and emerging trends. Bioengineering.

[80] Zhao Y., Laguna R.C., Zhao Y., Liu J.J., He X., Yianni J., Sarrigiannis P.G. (2018). A wavelet-based correlation analysis framework to study cerebromuscular activity in essential tremor. Complexity.

[81] Ortega A., Frossard P., Kovačević J., Moura J.M., Vandergheynst P. (2018). Graph signal processing: Overview, challenges, and applications. Proc. IEEE.

[82] Jang S., Moon S.E., Lee J.S. (2018). Graph Signal Representation of Eeg for Graph Convolutional Neural Network. https://openreview.net/pdf?id=Bk6Y0RR8M.

[83] Wu X., Zheng W.L., Li Z., Lu B.L. (2022). Investigating EEG-based functional connectivity patterns for multimodal emotion recognition. J. Neural Eng..

[84] Li X., Zhang Y., Tiwari P., Song D., Hu B., Yang M., Zhao Z., Kumar N., Marttinen P. (2022). EEG based emotion recognition: A tutorial and review. ACM Comput. Surv..

[85] Lee Y.Y., Hsieh S. (2014). Classifying different emotional states by means of EEG-based functional connectivity patterns. PLoS ONE.

[86] Petrantonakis P.C., Hadjileontiadis L.J. (2010). Emotion recognition from brain signals using hybrid adaptive filtering and higher order crossings analysis. IEEE Trans. Affect. Comput..

[87] Lan Z., Sourina O., Wang L., Liu Y. (2016). Real-time EEG-based emotion monitoring using stable features. Vis. Comput..

[88] Wang X.W., Nie D., Lu B.L. (2014). Emotional state classification from EEG data using machine learning approach. Neurocomputing.

[89] Sorkhabi M.M. (2014). Emotion detection from EEG signals with continuous wavelet analyzing. Am. J. Comput. Res. Repos..

[90] Mohammadi Z., Frounchi J., Amiri M. (2017). Wavelet-based emotion recognition system using EEG signal. Neural Comput. Appl..

[91] Rotem-Kohavi N., Oberlander T., Virji-Babul N. (2017). Infants and adults have similar regional functional brain organization for the perception of emotions. Neurosci. Lett..

[92] Olofsson J.K., Nordin S., Sequeira H., Polich J. (2008). Affective picture processing: An integrative review of ERP findings. Biol. Psychol..

[93] Bernat E., Bunce S., Shevrin H. (2001). Event-related brain potentials differentiate positive and negative mood adjectives during both supraliminal and subliminal visual processing. Int. J. Psychophysiol..

[94] Frantzidis C.A., Bratsas C., Papadelis C.L., Konstantinidis E., Pappas C., Bamidis P.D. (2010). Toward emotion aware computing: An integrated approach using multichannel neurophysiological recordings and affective visual stimuli. IEEE Trans. Inf. Technol. Biomed..

[95] Lang P.J., Bradley M.M., Cuthbert B.N. (1997). International affective picture system (IAPS): Technical manual and affective ratings. NIMH Cent. Study Emot. Atten..

[96] Nicolae I.E., Acqualagna L., Blankertz B. (2017). Assessing the depth of cognitive processing as the basis for potential user-state adaptation. Front. Neurosci..

[97] Nicolae I.E., Acqualagna L., Neagu G.M. (2018). Enhanced Classification Methods for the Depth of Cognitive Processing Depicted in Neural Signals. Univ. Politeh. Buchar. Sci. Bull. Ser. C-Electr. Eng. Comput. Sci..

[98] Craik F.I., Lockhart R.S. (1972). Levels of processing: A framework for memory research. J. Verbal Learn. Verbal Behav..

[99] Nicolae I.E., Acqualagna L., Blankertz B. (2015). Neural indicators of the depth of cognitive processing for user-adaptive neurotechnological applications. Proceedings of the 2015 37th Annual International Conference of the IEEE Engineering in Medicine and Biology Society (EMBC).

[100] Delorme A., Makeig S. (2004). EEGLAB: An open source toolbox for analysis of single-trial EEG dynamics including independent component analysis. J. Neurosci. Methods.

[101] Kim H., Luo J., Chu S., Cannard C., Hoffmann S., Miyakoshi M. (2023). ICA’s bug: How ghost ICs emerge from effective rank deficiency caused by EEG electrode interpolation and incorrect re-referencing. Front. Signal Process..

[102] Nicolae I.E. (2019). Advanced EEG Signal Processing with Applications in Brain-Computer Interfaces: Evaluating User Focused Paradigms for the Purpose of Enhancing Brain-Computer Interaction. Ph.D. Thesis.

[103] Winkler I., Haufe S., Tangermann M. (2011). Automatic classification of artifactual ICA-components for artifact removal in EEG signals. Behav. Brain Funct..

[104] Luck S.J. (2014). An Introduction to the Event-Related Potential Technique.

[105] Van Milligen B.P., Sanchez E., Estrada T., Hidalgo C., Brañas B., Carreras B., García L. (1995). Wavelet bicoherence: A new turbulence analysis tool. Phys. Plasmas.

[106] Grinsted A., Moore J.C., Jevrejeva S. (2004). Application of the cross wavelet transform and wavelet coherence to geophysical time series. Nonlinear Processes Geophys..

[107] König N., Steber S., Borowski A., Bliem H.R., Rossi S. (2021). Neural processing of cognitive control in an emotionally neutral context in anxiety patients. Brain Sci..

[108] Rubinov M., Sporns O. (2010). Complex network measures of brain connectivity: Uses and interpretations. Neuroimage.

[109] Stefano Filho C.A., Attux R., Castellano G. (2018). Can graph metrics be used for EEG-BCIs based on hand motor imagery?. Biomed. Signal Process. Control.

[110] Achard S., Bullmore E. (2007). Efficiency and cost of economical brain functional networks. PLoS Comput. Biol..

[111] Zhang J., Chen M., Zhao S., Hu S., Shi Z., Cao Y. (2016). ReliefF-based EEG sensor selection methods for emotion recognition. Sensors.

[112] Al-Nafjan A. (2022). Feature selection of EEG signals in neuromarketing. PeerJ Comput. Sci..

[113] Sakkalis V., Oikonomou T., Pachou E., Tollis I., Micheloyannis S., Zervakis M. (2006). Time-significant wavelet coherence for the evaluation of schizophrenic brain activity using a graph theory approach. Proceedings of the 2006 International Conference of the IEEE Engineering in Medicine and Biology Society.

[114] Wang G., Li J., Li Z., Wei M., Li S. (2016). Medial frontal negativity reflects advantageous inequality aversion of proposers in the ultimatum game: An ERP study. Brain Res..

[115] Jadhav N., Manthalkar R., Joshi Y. (2017). Effect of meditation on emotional response: An EEG-based study. Biomed. Signal Process. Control.

[116] Knyazev G.G., Slobodskaya H.R. (2003). Personality trait of behavioral inhibition is associated with oscillatory systems reciprocal relationships. Int. J. Psychophysiol..

[117] Bourdillon P., Hermann B., Guénot M., Bastuji H., Isnard J., King J.R., Sitt J., Naccache L. (2020). Brain-scale cortico-cortical functional connectivity in the delta-theta band is a robust signature of conscious states: An intracranial and scalp EEG study. Sci. Rep..

[118] Bae J.H., Choi M., Lee J.J., Lee K.H., Kim J.U. (2024). Connectivity changes in two-channel prefrontal ERP associated with early cognitive decline in the elderly population: Beta band responses to the auditory oddball stimuli. Front. Aging Neurosci..

[119] Roshanaei M., Norouzi H., Onton J., Makeig S., Mohammadi A. (2025). EEG-based functional and effective connectivity patterns during emotional episodes using graph theoretical analysis. Sci. Rep..

[120] Zhu G., Zong F., Zhang H., Wei B., Liu F. (2021). Cognitive load during multitasking can be accurately assessed based on single channel electroencephalography using graph methods. IEEE Access.

[121] Torres-Herraez A., Watson T.C., Rondi-Reig L. (2022). Delta oscillations coordinate intracerebellar and cerebello-hippocampal network dynamics during sleep. J. Neurosci..

[122] Aliramezani M., Farrokhi A., Constantinidis C., Daliri M.R. (2024). Delta-alpha/beta coupling as a signature of visual working memory in the prefrontal cortex. Iscience.

[123] Nácher V., Ledberg A., Deco G., Romo R. (2013). Coherent delta-band oscillations between cortical areas correlate with decision making. Proc. Natl. Acad. Sci. USA.

[124] Gómez-Lombardi A., Costa B.G., Gutiérrez P.P., Carvajal P.M., Rivera L.Z., El-Deredy W. (2024). The cognitive triad network-oscillation-behaviour links individual differences in EEG theta frequency with task performance and effective connectivity. Sci. Rep..

[125] Schlumpf Y.R., Nijenhuis E.R., Klein C., Jäncke L., Bachmann S. (2022). Functional connectivity changes in the delta frequency band following trauma treatment in complex trauma and dissociative disorder patients. Front. Psychiatry.

[126] Harper J., Malone S.M., Iacono W.G. (2017). Theta-and delta-band EEG network dynamics during a novelty oddball task. Psychophysiology.

[127] Su J., Zhu J., Song T., Chang H. (2023). Subject-independent eeg emotion recognition based on genetically optimized projection dictionary pair learning. Brain Sci..

[128] Liu J., Wu G., Luo Y., Qiu S., Yang S., Li W., Bi Y. (2020). EEG-based emotion classification using a deep neural network and sparse autoencoder. Front. Syst. Neurosci..

[129] Zhao J., Yang Y., An X., Liu S., Du H., Ming D. (2022). Auditory event-related potentials based on name stimuli: A pilot study. Front. Neurosci..

[130] Key A.P., Jones D., Peters S., Dold C. (2018). Feasibility of using auditory event-related potentials to investigate learning and memory in nonverbal individuals with Angelman syndrome. Brain Cogn..

[131] Kim K.H., Bang S.W., Kim S.R. (2004). Emotion recognition system using short-term monitoring of physiological signals. Med. Biol. Eng. Comput..

